# Wrist Extensor Muscle Fatigue During a Dual Task With Two Muscular and Cognitive Load Levels in Younger and Older Adults

**DOI:** 10.1177/00187208231218196

**Published:** 2023-12-06

**Authors:** Florestan Wagenblast, Thomas Läubli, Robert Seibt, Monika A. Rieger, Benjamin Steinhilber

**Affiliations:** 1 9188 Institute of Occupational and Social Medicine and Health Services Research, University Hospital Tübingen, Germany

**Keywords:** muscle fatigue, wrist extensors, cognitive task, tracking task, surface electromyography, maximum voluntary contraction force

## Abstract

**Objective:**

To examine the effect of concurrent physical and cognitive demands as well as age on indicators of muscle fatigue at the wrist.

**Background:**

There are few studies examining risk indicators for musculoskeletal disorders associated with work-related physical and cognitive demands that often occur simultaneously in the workplace.

**Methods:**

Twenty-four gender-balanced older and 24 gender-balanced younger (mean age 60 and 23 years) participants performed four 30 min dual tasks. Tasks differed by the muscular load level during force tracking: 5% and 10% of maximum voluntary contraction force (MVC) and concurrent cognitive demands on the working memory: easy and difficult. Muscle fatigue was assessed by MVC decline and changes in surface electromyography (increased root mean square: RMS, decreased median frequency: MF) at the extensor digitorum (ED) and extensor carpi ulnaris (EU).

**Results:**

A decline in MVC was found in all participants when tracking was performed at 10% MVC (mean ± SD: 137.9 ± 49.2 – 123.0 ± 45.3 N). Irrespective of age, muscular, or cognitive load, RMS increased (ED 12.3 ± 6.5 – 14.1 ± 7.0% MVE, EU 15.4 ± 7.6 – 16.9 ± 8.6% MVE) and MF decreased (ED 85.4 ± 13.6 – 83.2 ± 12.8 Hz, EU 107.2 ± 17.1 – 104.3 ± 16.7 Hz) in both muscles. However, changes in MF of EU tended to be more pronounced in the older group at higher cognitive and lower muscular load, without reaching statistical significance.

**Conclusion:**

Maximum voluntary contraction indicated no interaction between muscle fatigue, cognitive load, or age. However, the tendencies toward altered muscle activity due to an increase in cognitive load and older age suggest muscular adaptations while maintaining tracking performance during the onset of fatigue signs in the sEMG signal.

**Application:**

If the tendencies in muscle activity are confirmed by further studies, ergonomic assessments in industrial workplaces should consider cognitive load and age when describing the risk of musculoskeletal disorders.

## Introduction

Work-related musculoskeletal disorders (WRMSDs) cause sick leave, work disability, and early retirement. Moreover, the prevalence and severity of WRMSDs increase with age (Holmström & Engholm, 2003; van der Beek et al., 2017), which is of particular interest for an aging workforce, for example, in OECD (Organization for Economic Co-operation and Development) countries (OECD, 2017). Despite advances in research related to the underlying pathomechanisms (Barr et al., 2004), there is still no full explanation for the development of musculoskeletal disorders and their chronic manifestation (Barbe & Barr, 2006; Pelletier et al., 2015; Visser & van Dieën, 2006). This concerns, for example, pain perception and neurophysiological sensitization processes (Bonanni et al., 2022), insufficient recovery (Maganaris et al., 2017), or personal factors (Hunter, 2014). Epidemiological studies in the workplace have identified repetitive movements and heavy physical exertion as risk factors for WRMSDs (da Costa & Vieira, 2010; Kilbom et al., 1996; van der Molen et al., 2017). Besides these physical factors, high mental workload, including emotional, psychosocial, or cognitive demands, can also increase the risk of developing WRMSDs (Lang et al., 2012; Macfarlane et al., 2009; WHO, 2019). With the digitalization of work and the emergence of new technologies, cognitive demands may increase due to complex information processing, whereby simultaneous manual work is not necessarily reduced (Beer & Mulder, 2020). In addition, a growing on-demand production implies an increase in information density in the manufacturing of individualized products. These risk factors are associated with increased stress, especially fatigue, of exposed individuals (Macdonald, 2003; Meyer & Hünefeld, 2018; van der Windt et al., 2000).

Fatigue in the workplace is a significant problem in many industries, posing a threat to health and safety of employees or even others. In ergonomic research, muscle fatigue is considered a risk indicator of WRMSD (Council & Medicine, 2001) and is therefore commonly used to identify potential muscular overload in work activities.

However, a comprehensive definition of fatigue is difficult because it is a complex biological phenomenon and scientific disciplines have diverse perspectives. For this reason, Enoka and Duchateau (2016) proposed an integrative approach to study fatigue as a result of mental and physical factors that can interact and result in performance fatiguability and perceived fatiguability.

Regarding performance fatiguability, a decline in maximum force during isometric maximum voluntary contractions (MVC) indicates performance limitations of the muscles at maximum effort (Gandevia, 2001). The underlying limiting mechanisms involve domains of either contractile function or muscle activation that manifest at peripheral and central components located distal and proximal to the neuromuscular junction sites, respectively (Gandevia, 2001). Contractile function and muscle activation can also affect the surface electromyography (sEMG) signal that represents the myoelectrical output of a muscle. According to Luttmann et al. (1996), a combined assessment of amplitude and frequency domain parameters reflects changes in neuromuscular activation during performance that are linked to the development of muscle fatigue. However, according to Enoka and Duchateau (2016), it cannot be excluded that the manifestation of performance fatigue is influenced by factors that contribute to perceived fatigue. Therefore, complementary assessment of perceived effort may reveal changes in regulatory processes regarding effects of individual mental (e.g., motivation and pain) and physiological (e.g., core temperature and hydration) states on the development of fatigue (Enoka & Duchateau, 2016; Smirmaul Bde, 2012). Gandevia (2001) gives a good overview of how signs of muscle fatigue can be detected.

Existing theories on the contribution of central factors to regulatory processes that are linked to fatigue development suggest that neural drive to muscles is controlled by performance feedback and subconsciously by maintaining homeostasis (Blain et al., 2016; St Clair Gibson & Noakes, 2004). There is evidence that neural drive can be influenced via sensory afferents to cerebral cortical regions associated with executive functions and is presumably modulated by fatigue perception and effort regulation (de Morree et al., 2014; Taylor et al., 2016). Tasks with high cognitive demands on effort regulation, like dual task situations that require redirection and appropriate allocation of attention to allow for adequate motor response, could affect neural drive to the muscles (Bray et al., 2012) and contribute to fatigue. While most studies found higher perceived effort, and endurance decrements due to additive mental demands, the results on the muscle fatigue indicators MVC and sEMG activity, are inconclusive (Chatain et al., 2019; Lorist et al., 2002; Mehta & Agnew, 2012; Mehta et al., 2012; Temprado et al., 2015; Yoon et al., 2009). In dual task experiments at the wrist muscles, sustained isometric contractions at 30% MVC led to a decreased endurance time in older individuals without affecting MVC (Shortz & Mehta, 2017). Lower amplitude of electromyographic activity through additive cognitive load was found in a group of younger individuals during intermittent isometric contractions at 65% MVC (but not at 5% MVC) and was interpreted as an early indicator of altered muscle capacity (Mehta et al., 2012).

With age, limitations in neural activation of muscles during voluntary contractions may be caused by changes in the central nervous system (Yoon et al., 2008). Changes concern, for example, dysfunction of the cortical neuron or reduced excitability of the motor cortex and motoneuron pool (Hunter, 2016; Yoon et al., 2008). In particular, (fine) motor performance has been found to be increasingly controlled by cognitive brain processes of motor control from about age 60, which has been attributed to structural and functional regress in the motor cortical regions (Pereira et al., 2018; Seidler et al., 2010), leading to higher demands on attentional resources. It is also known that cognitive function changes with age, the processing of and reaction to information is negatively affected. Performance impairments are most apparent in complex attention tasks (Allman & Rice, 2002). In studies examining brain activity in older adults during dual tasks involving memory functions, less activity was observed in some brain areas on the one hand, and signs of an over recruitment of activity in those who performed better on the other (Grady, 2008). Pereira et al. (2015) pointed out that during cognitive performance interference can increase at corticospinal connections originating from the prefrontal cortex. Taken together, age-related neurophysiological processes that limit motor and cognitive function might contribute to an increased effort as compensation to maintain task performance and might result in an accelerated, centrally induced muscle fatigue development.

However, few ergonomic studies have examined the development of muscle fatigue considering an aging workforce, as well as occupational demands that require concurrent fine motor skills and basic cognitive functions. In this context, it is important to consider muscular and cognitive demand levels occurring in real work environments.

The aim of this study was to investigate the influence of two levels of muscular and cognitive load during a 30 min dual-task paradigm on measurements associated with muscle fatigue in two age groups (younger and older German adults of working age between 18 and 67 years old). Motor demands were simulated using a tracking task with a varying low submaximal target force at levels of 5% or 10% MVC on average, comparable to wrist muscle activity during industrial manual work (Nordander et al., 2004). This required fine motor adaptations during intermittent isometric muscle contraction, interrupted by short breaks, comparable to the handling of tools. Cognitive demands were manipulated through the workload of the working memory, which is considered a fundamental cognitive function required to process, update, and store any type of information. Its capacity is limited by the amount of information and attentional resources (Baddeley, 2010). Muscle fatigue outcomes were assumed to increase due to higher muscular and cognitive load, with the latter effect possibly moderated according to age group.

## Methods

### Participants

Participants were recruited through mailing lists of the University Hospital Tübingen and public postings. The inclusion criteria were no self*-*reported acute pain or musculoskeletal complaints at the upper extremities, no neurological or psychological diseases, a body mass index between (BMI) 19–31 kg/m^2^, age 18–27 or 50–67 years, and no acute medication regarding β-blockers, analgesics, antipsychotics, antidepressants, anticonvulsants, or anxiolytics. The lower limit for the older age group was based on definitions that classify workers as older when they are 45 or 55 years of age (Rix, 2001; WHO, 1993). Consistent with the full-factorial study design and a balanced gender ratio (12 women, 12 men), recruitment was only stopped when the data sets of 24 participants in each age group could be considered for further analysis. All participants received financial compensation and provided written informed consent prior to study participation. The study was advised by the ethics committee of the Medical Faculty of the University of Tübingen (274/2018BO2) and was conducted according to the principles of the Declaration of Helsinki (version 2013, Fortaleza). 

### Dual Task and Sample Size

The dual task was presented on a computer screen (S1931, resolution 1280 × 1024, Eizo Corp, JP) using self-developed software (Force&Brain, University Hospital Tübingen, DE) and included a tracking task and a concurrent cognitive task.

**Figure 1.**
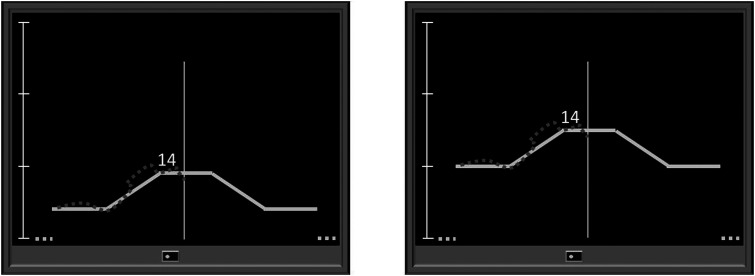
Drafts of the visual representation of the dual task on the computer screen with an average of 5% MVC (left) and 10% MVC (right). Fair solid line: target force template with five consecutive 5 s phases: (1) constant force level, (2) increasing force level with a slope of 1% MVC per 1 s, (3) constant force level, (4) decreasing force level with a slope of 1% MVC per 1 s, (5) constant force level; fair dashed line: 5 s pause; dark dashed line: feedback of applied force; centered vertical line: marks current target and applied force; scale of the target force as a percentage of the maximum force (% MVC) with the maximum at 30% MVC; random two-digit number close to centered vertical line of the cognitive task.

During the tracking task, participants reproduced the course of a trapezoid target force using isometric dorsal wrist extension of the dominant hand, while visual feedback on the applied force was provided. The target force template moved continuously from the right to the left side of the screen, followed by a 5 second pause. The trapezoid pattern consisted of five 5 second phases, three with constant, one with increasing, and one with decreasing target force. This pattern and the subsequent 5 second pause recurred every 30 s and is subsequently referred to as 30 s period. On average, the two levels of muscular load were performed at 5% MVC, ranging from 0–9% MVC (5%MVC), or at 10% MVC, ranging from 0–15% MVC (10%MVC). Across force levels, the displayed scale of the target force was kept constant with a maximum of 30% MVC (Figure 1).

For the cognitive tasks, random two-digit numbers were displayed close to the target force template for 1 s and replaced after another second without a number (Figure 1). The cognitive tasks were the same for each participant and included a lower cognitive load level, in which participants had to detect a given number (0-back task), and a higher cognitive level, in which participants had to detect each number that was displayed two numbers before (2-back task). To confirm a target number, participants had to press a button with their nondominant hand as quickly as possible. The 0-back task can be considered a two-alternative forced choice task that places fewer demands on the working memory than the 2-back task, which is a variant of an n-back task that requires motor responses to visual stimuli (Gajewski et al., 2018).

Four experimental conditions were applied. A within-subject full-factorial design was used to compensate for carryover effects. Based on the four conditions, the sample size was set at 24 participants per age group to ensure that each participant completed the experimental tasks in one of the 24 possible orders. Participants within the two age groups were assigned randomly to one of the 24 possible order sequences of the four conditions.

### Procedure

The study comprised three appointments for each participant spread over three days with 2–7 days between appointments.

**Figure 2.**
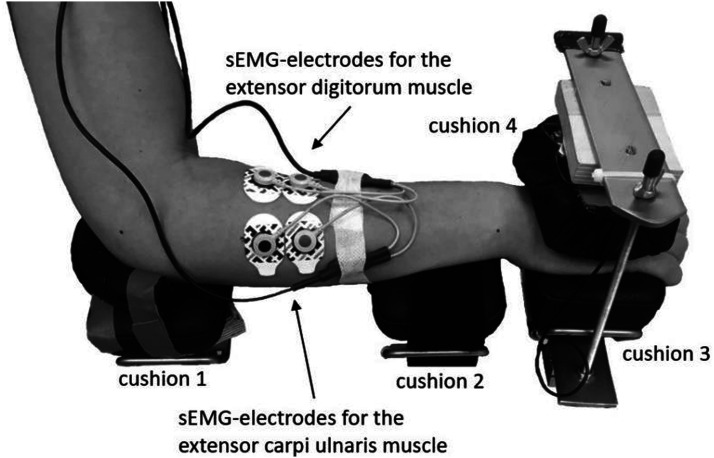
Adjustable armrest of the experimental setup and electrode placement at the wrist extensors of the dominant arm. Cushion 1 below the elbow; cushion 2 below the wrist (the outer edge aligned with the ulnar styloid process); cushion 3 below the hand (the outer edge aligned with the distal end of the metacarpal bones and the wrist flexed at approximately 5°); cushion 4 at the back of the hand (aligned with cushion 3 under constant contact pressure of 15–20 N).

On the first appointment, the experimental setup was adjusted to participants’ body size and used for each subsequent appointment. Participants were seated on a chair with a fixed backrest. A hip and knee flexion angle of approximately 95° was ensured. The dominant arm was placed on an armrest with four adjustable cushions equipped with force sensors (Figure 2). Both arms were placed at approximately 40° abduction, while shoulders were relaxed. The elbows were bent at approximately 95°. A computer screen was placed 1.5 m in front of the participants, with the upper edge set at eye level. After a general familiarization with the dual task, participants practiced each condition for approximately 2.5 min. Finally, participants were asked to refrain from sports and alcohol at least one day prior to the measurements, and to abstain from smoking, caffeine, or other stimulants on the day of the subsequent appointments.

The second and third appointment started at the same time of day (±1 h) for each participant and followed the same procedure including two measurements during each appointment. At both appointments, participants received instructions about the procedure and the dual task using a standardized slideshow. Based on the assumptions that lower muscular load leads to faster muscular recovery and that the 2-back task is more prone to adaptation effects, participants practiced the dual task with lower muscular and higher cognitive load for 5 min.

**Figure 3.**
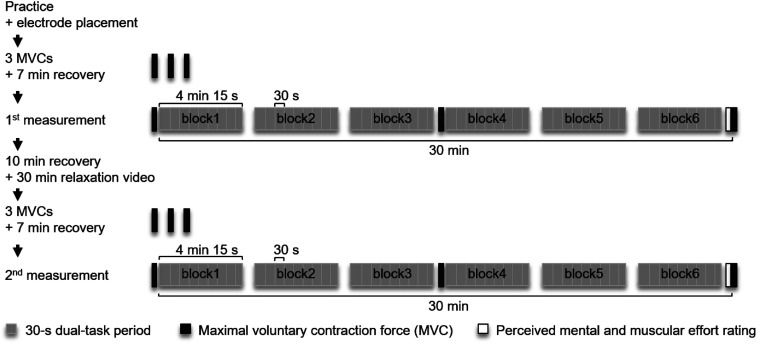
Procedure on the second and third appointment including two measurements.

Ten minutes before the first measurement, participants performed three MVC measurements of the wrist extensors followed by 7 min of recovery. The subsequent measurement lasted for 30 min and was divided into six blocks with dual-task durations of 4 min 15 s each. During the 45 s between blocks, participants continued tracking without simultaneously following the cognitive task, and instead answered questions about their perceived effort which are not included in the present analysis. Between block3 and block4, tracking was replaced by an MVC measurement. After completing the first measurement, participants remained seated in the experimental setup for 10 min. They then watched a relaxation video (submarine or forest theme) while seated in a comfortable armchair for another 30 min before the procedure was repeated with a different experimental condition (Figure 3).

### Control Measurements

Perceived effort and force steadiness during the tracking task were recorded to verify differences in perceived muscular and cognitive load and fine motor performance.

#### Perceived Effort

To quantify the perceived effort, participants first rated the muscular effort of their dominant arm and then their mental effort on an 11-point numerical rating scale (Lampropoulou & Nowicky, 2012; Yeo & Neal, 2004) verbally at the end of each measurement while the tracking task continued. A value of ten represented the greatest effort imaginable, whereas zero represented no effort.

#### Force Steadiness

As a measure of fine motor performance that could affect muscle activity over time and thus muscle fatigue outcomes (Visser et al., 2003), force steadiness was calculated as the standard deviation of applied force during the tracking task (SD [N]). The applied force was recorded using a high-resolution force sensor (Model 615 Tedea-Huntleigh, accuracy .1%, Vishay Precision Group, USA) placed below cushion 3 (Figure 2). The sensor was coupled with two amplifiers (DMS-Messverstärker, THUMEDI GmbH & Co. KG, Germany and AMS42-LAN16fx, BMC Messsysteme GmbH, Germany) and the signal was sampled and stored at 8 Hz.

#### Fatigue Measurements

##### Maximum Voluntary Contraction Force

MVC [N] was measured by a force sensor (KM38-1 kN, accuracy 1%, ME-Meßsysteme, Germany) installed above cushion 4 and connected to an amplifier (AMS42-LAN16fx, BMC Messsysteme GmbH, DE). It was originally intended to measure MVC using the sensor below cushion 3 during an MVC; however, test measurements showed that participants might exert pressure instead of traction on the sensor below cushion 3 during an MVC measurement. The signal during isometric muscle contractions of the wrist extensors was sampled and stored at 8 Hz using self-developed software (Force&Brain, University Hospital Tübingen, Germany). During the MVC measurements, participants sat in the experimental setup and extended their wrist at maximum strength for about 5 s by pressing the back of their hand against cushion 4. They received no visual feedback about the applied force but were verbally encouraged to exert maximum effort. Before the measurements, three MVC measurements with 45 s rest in between were recorded to determine the target force levels of the tracking task. Three additional MVC measurements were carried out before, in the middle of, and after the dual task to determine muscle fatigue, which was defined as decrease from start to end.

##### Electromyographic Muscle Activity

Skin was prepared using abrasive paste (Nuprep, Weaver and Company, USA). Bipolar surface electrodes (H93SG, Covidien, Germany) were attached to the extensor digitorum (ED) and the extensor carpi ulnaris (EU) parallel to the muscle fibers according to the recommendations from Crisswell (2011) with an electrode distance of 25 mm. A neutral electrode was placed over the seventh cervical vertebrae.

SEMG signals were sampled at 4096 Hz, analyzed, and stored using a combined data analyzer and logger (PS12-II, THUMEDI GmbH & Co. KG, Germany). The root mean square (RMS [μV]) and the median frequency (MF [Hz]) were real-time calculated from the power spectrum and stored by the device. Signs of muscle fatigue were defined as an increase in RMS and a decrease in MF (Basmajian & De Luca, 1985).

### Data Processing and Statistical Analyses

The SD, median RMS, and median MF were calculated from the middle 3 s of each upper 5 s phase of the trapezoid during 30 s periods to limit interferences in the sEMG signal due to fluctuating or very low loads (Gonzalez-Izal et al., 2012). The target force was constant at 9% (5%MVC) or 15% MVC (10%MVC). The first 30 s periods of the remaining blocks were excluded to minimize adaptation effects. Exclusion criteria for 30 s periods included interference with EMG signals due to technical problems or procedural violations, that is, speaking, eyes closed, apparent movements, or other distractions. The mean applied force during the 3 s had to be within ±15% of the target force to ensure constant muscular load, and missing MF data due to low muscle activity had to be <50%. This led to the exclusion of <1% of all 30 s periods. The remaining RMS data was normalized to the 90^th^ percentile of the highest RMS values of three consecutive seconds during the first three MVC measurements at each appointment. The MVC-normalized muscle activity was calculated as a percentage of the maximum electrical muscle activity [% MVE]. Then, SD, median RMS, and median MF were averaged for each 5 min block.

Separate linear mixed models were applied to SD, MVC, RMS, and MF. Fixed factors included age group (older, younger) and crossed factors were muscular load (5%MVC, 10%MVC) and cognitive load (0-back, 2-back task), as well as time course (MVC: start, end; SD, RMS, and MF: block1, block6). To account for the crossed-nested data structure, two random effects were considered: day of appointment within participant and participant within age group. Due to limited prior knowledge of the course of eventual muscle fatigue and to reduce statistical comparisons, we decided to include only start and end points of the exposures in the statistical model.

For muscle fatigue outcomes (MVC, RMS, MF), a complementary exploratory analysis was performed to further minimize statistical comparisons by analyzing relative differences between start and end as the percentage change using the formulas:

(1) $${\text{MVC}}_{\text{REL}-\text{DIFF}}=\left(\text{start}\;–\;\text{end}\right)\;/\;\text{start}\;x\;100$$

(2) $${\text{RMS}}_{\text{REL}-\text{DIFF}}\;\text{and}\;{\text{MF}}_{\text{REL}-\text{DIFF}}=\left(\text{block}1\;–\;\text{block}6\right)\;/\;\text{block}1\;x\;100.$$

Separate linear mixed models were performed for these dependent variables (MVC_REL-DIFF_, RMS_REL-DIFF_, MF_REL-DIFF_) and perceived muscular and mental effort, including the fixed effects of age group, muscular load, and cognitive load, as well as the random effects mentioned above. Post hoc comparisons were carried out using Tukey’s Honestly Significant Differences.

Height, weight, maximum force (maximum of the first three MVCs, averaged over both appointments), and MVC-normalized RMS during block1 of the wrist extensors were compared to identify differences between age groups using unpaired t-tests. The comparisons of MVC-normalized RMS at the beginning of the dual task allowed us to control for group differences in muscular effort that could potentially influence the development of muscle fatigue outcomes, as muscular load levels of dual task conditions were individually adjusted for maximum force. To do so, the MVC-normalized median RMS of block1 was averaged across all conditions.

Prior to the statistical analysis, variables were verified for normal distribution visually and using skewness and kurtosis. Although not fully given, data were treated as normally distributed. This was justified by the robustness of the linear mixed models regarding a violation of the normality assumption (Harwell et al., 1992; Lumley et al., 2002). An additional analysis with log-transformed data led to the same statistical results. The level of significance was set at α = .05. All statistical analyses were conducted with the software JMP 14 (SAS Inc, USA).

## Results

### Participants

Of the 54 participants, six were excluded from data analysis: three due to discomfort on the first appointment, one BMI >31, one unavailable for a third appointment, one had closed eyes during tracking on day three. T-tests of anthropometric characteristics, maximum force, and MVC-normalized muscle activity during block1 showed no significant differences between age groups (Table 1).

**Table 1.** Unpaired t-Tests Applied to Anthropometric Characteristics, Maximum Force, and Muscle Activity During block1.

	Age Group	Means (SD)	*p-*values
Height (cm)	Older	168.7 (7.0)	.063
	Younger	173.4 (9.5)	
Weight (kg)	Older	69.3 (9.5)	.903
	Younger	69.7 (9.3)	
Maximum force (N)	Older	131.4 (49.6)	.866
	Younger	143.2 (45.2)	
RMS extensor digitorum (% MVE)	Older	12.7 (5.7)	.598
	Younger	11.9 (4.6)	
RMS extensor carpi ulnaris (% MVE)	Older	15.9 (4.8)	.549
	Younger	14.9 (7.1)	

Means and standard deviation (*SD*) in brackets for height, weight, maximum voluntary contraction force of the wrist extensors derived from the maximum voluntary contractions (MVC) at the start of each measurement, and root means square (RMS) of wrist extensors during block1 normalized to RMS during MVC measurements, of older and younger participants, including 12 women and 12 men in each group.

### Control Measurements

#### Perceived Effort

Perceived muscular effort showed a significant interaction between age group and muscular load (*F* (*df* = 1, *df of residuals* = 134.8) = 45.3, *p* < .001) (Supplementary material 1). Post hoc comparisons indicated that the older group rated the muscular effort significantly higher when the tracking task was performed at 5% MVC compared to the younger group (mean older: 5.4, younger: 3.6, *p* = .006). At 10% MVC there was no significant group difference (mean older: 6.1, younger: 6.7, *p* = .852) (Supplementary material 2).

Analogously, there was a significant interaction between age group and cognitive load (*F* (1, 137.7) = 19.8, *p* < .001) regarding ratings of perceived mental effort (Supplementary material 1). Post hoc comparisons revealed that the older group rated the mental effort higher after the 0-back task conditions compared the younger group (mean older: 5.4, younger: 2.8, *p* < .001). For the 2-back conditions there was no significant group difference (mean older: 7.6, younger: 6.6, *p* = .158) (Supplementary material 2).

#### Force Steadiness

For SD, time course and muscular load had a statistically significant interaction (*F* (1, 275.0) = 5.8, *p* = .017), with a statistically significant increase of 23% from block1 to block6 in 10% MVC conditions (mean .40–.49 N, *p* < .001) but not in 5% MVC conditions. Time course had no statistically significant interaction effect with age group or any other factor (Supplementary material 3, 4).

### Muscle Fatigue Measurements

#### Maximum Voluntary Contraction Force

**Figure 4.**
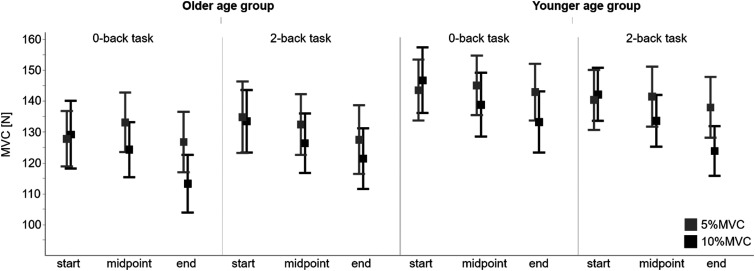
Time course of the maximum voluntary contraction force of the wrist extensors. Means and standard error of the maximum voluntary contraction force measured before the start, at midpoint, and at the end of the dual task comparing muscular load conditions (5%MVC grey, 10%MVC black) with respect to age groups (older left, younger right) and cognitive load conditions (0-back task left, 2-back task right).

The statistical analysis of MVC revealed a significant interaction of muscular load and time course (*F* (1, 272.6) = 15.3, *p* < .001), with post hoc comparisons indicating a statistically significant MVC decrease of 12% in the 10% MVC conditions (mean 137.9–123.0 N, *p* < .001), without changes in 5% MVC conditions. There was no statistically significant association between age group and cognitive load (Figure 4, Table 2, Supplementary material 5). Complementary exploratory analysis of relative differences also revealed no statistically significant effects of age group or cognitive load.

**Table 2.** Mixed Analyses of Variance Applied to Maximum Voluntary Contraction Force.

Effect on Maximum Voluntary Contraction Force	MVC		
	*df*, *df* Residuals	*F-*values	*p-*values
Age group	1, 46.0	.8	.365
Muscular load	1, 322.0	4.9	.028^a^
Cognitive load	1, 322.0	<.1	.841
Time course	1, 272.6	32.7	< .001^a^
Age group*muscular load	1, 322.0	<.1	.957
Age group*cognitive load	1, 322.0	3.0	.082
Age group*time course	1, 272.6	<.1	.899
Muscular load*cognitive load	1, 322.0	1.7	.194
Muscular load*time course	1, 272.6	15.3	< .001^a^
Cognitive load*time course	1, 272.6	.5	.467
Age group*muscular load*cognitive load	1, 322.0	.2	.695
Age group*muscular load*time course	1, 272.6	.5	.471
Age group*cognitive load*time course	1, 272.6	.1	.731
Muscular load*cognitive load*time course	1, 272.6	.3	.573
Age group*muscular load*cognitive load*time course	1, 272.6	1.1	.297

Results of the maximum voluntary contraction force of the wrist extensors parameterized maximum force applied during maximum voluntary contractions (MVC) considering the factors age group (older, younger), muscular load (5%MVC, 10%MVC), cognitive load (0-back, 2-back task), and time course (start, end).

^a^Statistically significant effect (α = .05).

#### Electromyographic Muscle Activity

**Figure 5.**
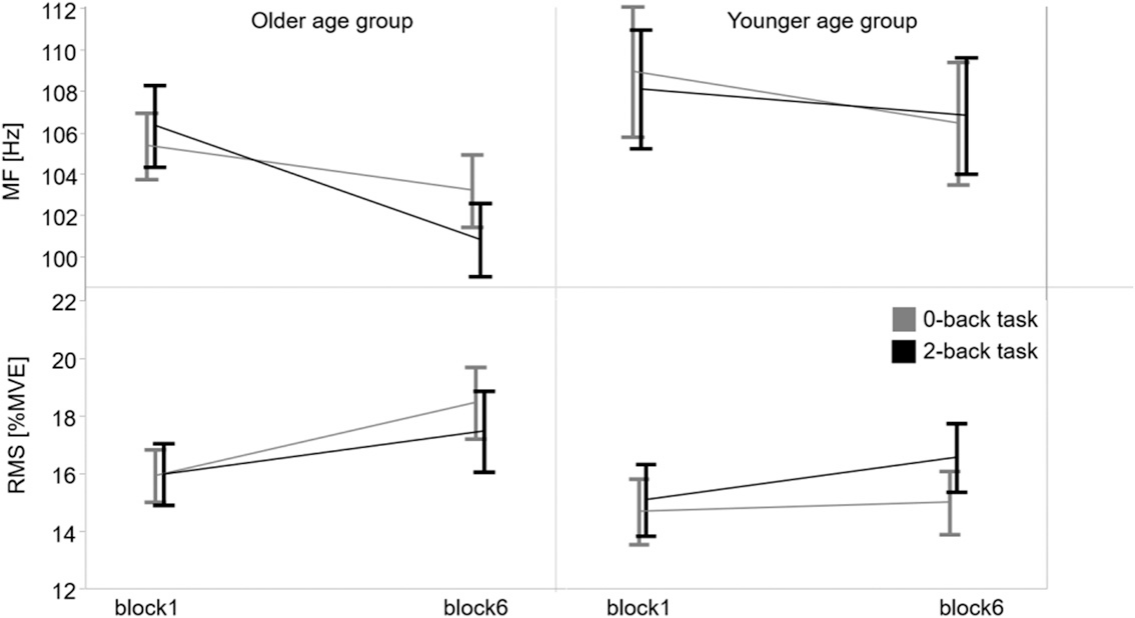
Development of median frequency and root mean square of the extensor carpi ulnaris from block1 to block6. Means and standard error of the median frequency (MF above) and the normalized root mean square (RMS below) of the extensor carpi ulnaris measured by surface electromyography during the tracking tasks in block1 and block6 comparing cognitive load conditions (0-back task grey, 2-back task black) with respect to age groups (older left, younger right).

Extensor carpi ulnaris showed a statistically significant 2.7% decrease in MF (mean 107.2–104.3 Hz, *F* (1, 274.2 = 21.0), *p* < .001) and a 9.7% increase in RMS (mean 15.4%–16.9% MVE*, F* (1, 274.1) = 14.9, *p* < .001) over time, irrespective of muscular load and cognitive load or age group. The threefold interaction term age group*cognitive load*time course came close to statistical significance for MF (*F* (1, 274.2) = 3.3, *p* = .069) (Figure 5; Table 3). Complementary exploratory analysis revealed a statistically significant two-fold interaction of age group and cognitive load on MF_REL-DIFF_ (*F* (1, 134.6) = 5.2, *p* = .024) and RMS_REL-DIFF_ (*F* (1, 137.7) = 4.7, *p* = .032). Thus, there seemed to be a tendency of a stronger MF decrease in the older age group when higher cognitive load was applied, with RMS tending to increase less (Figure 5, Table 3, Supplementary material 6, 7, 8, 9). However, post hoc comparisons did not reach statistical significance and it therefore remains a tendency that needs further exploration.

**Table 3.** Four-Factor Mixed Analyses of Variance Applied to Median Frequency and Root Means Square of the Extensor Carpi Ulnaris.

Effect on Muscle Activity of the Extensor Carpi Ulnaris	MF			RMS		
	*df*, *df* Residuals	*F-*values	*p-*values	*df*, *df* Residuals	*F-*values	*p-*values
Age group	1, 46.0	0.7	.421	1, 46.0	.8	.3882
Muscular load	1, 317.8	9.7	.002^a^	1, 316.7	131.7	< .001^a^
Cognitive load	1, 317.8	.4	.522	1, 316.7	.1	.720
Time course	1, 274.2	21.0	< .001^a^	1, 274.1	14.9	< .001^a^
Age group*muscular load	1, 317.8	<.1	.782	1, 316.7	7.5	.007^a^
Age group*cognitive load	1, 317.8	<.1	.953	1, 316.7	1.3	.260
Age group*time course	1, 274.2	2.4	.124	1, 274.1	2.2	.140
Muscular load*cognitive load	1, 317.8	.4	.553	1, 316.7	.1	.763
Muscular load*time course	1, 274.2	2.7	.103	1, 274.1	.2	.626
Cognitive load*time course	1, 274.2	.7	.400	1, 274.1	<.1	.941
Age group*muscular load*cognitive load	1, 317.8	<.1	.810	1, 316.7	.1	.743
Age group*muscular load*time course	1, 274.2	.6	.451	1, 274.1	.9	.340
Age group*cognitive load*time course	1, 274.2	3.3	.069	1, 274.1	2.1	.150
Muscular load*cognitive load*time course	1, 274.2	2.0	.164	1, 274.1	<.1	.820
Age group*muscular load*cognitive load*time course	1, 274.2	.5	.499	1, 274.1	<.1	.909

Results of the electromyographic muscle activity of the extensor carpi ulnaris parameterized as the median frequency (MF) and the normalized root mean square (RMS) considering the factors age group (older, younger), muscular load (5%MVC, 10%MVC), cognitive load (0-back, 2-back task), and time course (block1, block6).

^a^Statistically significant effect (α = .05).

For ED, main effects of time course indicated a statistically significant MF decrease (*F* (1, 274.1) = 15.1, *p* < .001) and RMS increase (*F* (1, 274.5) = 26.8, *p* < .001), independent of the other factors (Supplementary material 10). MF decreased by 2.6% (mean 85.4–83.2 Hz) and the RMS increased by 14.6% (mean 12.3–14.1 % MVE) from block1 to block6 (Supplementary material 11, 12). The complementary exploratory analysis of RMS_REL-DIFF_ and MF_REL-DIFF_ confirmed that developing muscle fatigue was independent from the other factors.

## Discussion

The main findings suggest that signs of muscle fatigue, measured by an MVC decline, could only be detected when the dual task was performed at higher muscular load (10% MVC), while sEMG also revealed signs of muscle fatigue at the lower muscular load (5% MVC). Furthermore, the sEMG data of EU may point towards a tendency of the older to be more susceptible to muscle fatigue during higher cognitive load.

The two control parameters, perceived muscular effort and perceived mental effort, were used to ensure that the assumption of distinct muscular and cognitive load levels was met. This was subjectively confirmed by higher ratings following conditions with higher loads. Overall, perceived effort was rated similarly to that in real occupational settings (Burgel & Elshatarat, 2019). The control parameter, force steadiness, decreased over time independently of age group and cognitive load, suggesting no potentially confounding differences of muscle activity due to variations in the course of precision performance between these factors.

### Influence of Muscular Load

The assumption that higher muscular load increases signs of muscle fatigue was confirmed by a larger MVC decline when the tracking task was performed at 10% MVC. This suggests performance fatiguability of the wrist extensors due to impairments of the nervous system during low sustained muscle activity (Westerblad et al., 2010), as the nervous system controls the exerted force during maximum voluntary contractions, or local phenomena like a suboptimal blood flow within muscles (Wan et al., 2017). These results are consistent with the higher perceived muscular effort at the end of the 10% MVC conditions, but differ from those of the sEMG parameters, which showed signs of muscle fatigue independently of muscular load, even during tracking at 5% MVC under intermittent contractions with 5 s pauses every 25 s. This may be related to the possibility that during intermittent, prolonged low-intensity muscle contractions, changes in sEMG parameters may reflect more neuromuscular adaptations to maintain force production rather than necessarily being associated with functional losses in maximum strength (Taylor et al., 2016). Further, MVC measurements in the middle of the dual task may have triggered de-recruitment of muscle units that prevented differences in signs of muscle fatigue in the sEMG signal at lower loads, following the Cinderella hypothesis that low-threshold motor units are overloaded by permanent stereotypic activation (Sjogaard & Sogaard, 1998). Theoretically, these motor units do not rotate during sustained or highly repetitive low-load tasks that would allow metabolic recovery (Bawa et al., 2006). Thus, additional motor units must be recruited to maintain task performance or force production, resulting in increased RMS in the sEMG signal.

### Influence of Age Group and Cognitive Load

Based on the MVC results, higher cognitive load did not promote the manifestation of increased signs of muscle fatigue in either of the two age groups. Similarly, Chatain et al. (2019) found no difference in MVC decrements or endurance time in younger men (mean 23 years) after a muscle fatiguing dual task when they compared a 1-back with a 2-back task. However, the control condition without a cognitive task differed in endurance time, but not in MVC. Yoon et al. (2009) also found no significant differences in the decrease in MVC in either men or women (mean 23 years) after sustained fatiguing contraction of the elbow flexors at 20% MVC, with and without cognitive stress (mental-mathematical task). Pereira et al. (2015) compared three fatiguing conditions of sustained isometric contraction at 20% MVC with no, low, and high cognitive demands until task failure for older women and older men (mean 71 years). In older women, but not in men, time to task failure was shorter when cognitive stress was added, but neither condition had an effect on MVC.

The sEMG results of ED showed muscle fatigue independently from cognitive load and age, which is consistent with the results of Pereira et al. (2015) in older adults. Mehta and Agnew (2012) also observed in younger men and women (mean 22 years) no change in fatigue effects in the sEMG signal when mental workload was added, but in endurance time when participants performed intermittent isometric contractions at 35% MVC. The complementary exploratory analysis on MF and RMS showed a significant interaction of age and cognitive load for EU. The tendency of a stronger decrease in MF in the older group when the higher cognitive load was applied supports the assumption that older participants may be more susceptible to fatigue when cognitive capacity is limited during a dual task. One explanation could be that at a very low muscular load of about 5% MVC, muscle fatigue originates to a greater extent from the central nervous system and neural drive (Smith et al., 2007). These central fatigue mechanisms seem to intensify with higher mental demands, in particular with age-related neural changes (Pereira et al., 2015; Yoon et al., 2008). In contrast, Guzman-Gonzalez et al. (2020) found a lower decreased MF at the flexor carpi radialis in younger adults (mean 22 years) due to dual-task demands compared to a single task. Their dual task consisted of hand gripping at constant 50% MVC and a subtraction task. They suggested that higher force steadiness during the single task led to a more demanding strategy of the neuromuscular system.

The sEMG results might also be interpreted in the context of different motor control strategies, especially when dynamic motor tasks are combined with additional cognitive load in a dual task. Kao et al. (2018), for instance, studied walking under local fatigue with concurrent cognitive challenges and demonstrated that different compensatory strategies are initiated to counteract the disturbances of equilibrium. These mechanisms altering sensory input, as well as motor output, are believed to be initiated and controlled on a cortical level (Paillard, 2012). In a target-oriented tracking task, similar strategies, including time-dependent inhomogeneous activation patterns of muscle units, intramuscular regions, and synergistic muscles (Contessa et al., 2018; Holtermann et al., 2010), might have led to a higher inter-individual variability in sEMG signals of individual muscles.

### Strengths and Limitations

One strength of this study is the sample size of 48 participants, which was much smaller in previous comparable studies (Chatain et al., 2019; Pereira et al., 2015).

Limitations concern possible effects of sex on muscle fatigue, which was not part of the research question, but could have contributed to greater variability in muscle fatigue development within age groups. These differences in fatigability between men and women are task specific and can partly be explained by physiological differences (Hunter, 2014, 2016; Kavanagh et al., 2020). Due to the limited sample size and potential bias from a noncontrolled order effect regarding men and women, we refrained from sex-specific subgroup analyses. However, although not reported in the result section, we repeated our complementary statistical analysis by adding the variable sex as a covariate in order to control for possible sex effects on the fatigue indicators. The results of these covariance analyses showed no statistically significant influence of sex.

Intensity and length of physical tasks were found to be important determinants in predicting physical performance decrements due to cognitive fatigue respectively load (Van Cutsem et al., 2017). Therefore, a stronger distinction between force levels or a longer task duration might have led to more differentiated signs of muscle fatigue (in the sEMG signal) between experimental conditions concerning effects of cognitive load and age. However, the relatively low tracking force levels accounted for physical loads that occur in industrial workplaces (Birch et al., 2000; Laursen et al., 2001; Nordander et al., 2004).

The acquisition of the mental effort is another point of concern. Although it was recorded during the course of the test, it only represents a global measure of cognitive exertion due to the methodology used (subjective rating). A dedicated analysis of a possible shift in attention within the dual task was therefore not possible.

Finally, we did not assess participants’ physical fitness level, which could have an impact on muscle fatigue. However, we recorded participants’ MVC of the target muscles, which was comparable between the older and younger group (Table 1) and is considered a factor influencing local muscle strength endurance and muscle fatigue (Enoka & Duchateau, 2008; Tibana et al., 2021). In addition, muscular effort at the beginning of dual task conditions (MVC-normalized muscle activity during block1) was also similar between the age groups (Table 1).

## Conclusion

With rather low muscle force requirements typical for industrial work scenarios, older age and higher cognitive loads may be factors associated with changes in muscular activation patterns over time, which potentially increase fatigue development, when motor performance is to be maintained. To confirm this trend and to find out more about its relevance, further research is needed that more closely replicates occupational workloads and conditions including longer exposures or more complex motor tasks.

Presumably, in scenarios with very low prolonged muscle activity, changes in sEMG parameters may be more sensitive than an MVC decline to identify signs of muscle fatigue because neuromuscular adaptations could be initiated before a strength loss at maximum effort is detectable. This may be of particular relevance for ergonomic research, which aims to identify cues for ergonomic workplace design. In general, the results of the present study underline the need of further research on older workers and potential health hazards due to changing cognitive requirements in the scope of the digitalization of work.

## Key Points

- A decline of maximum voluntary contraction force of the wrist extensors was found when tracking was performed at 10% of maximum voluntary contraction force, but not at 5%.
- Surface electromyography showed signs of muscle fatigue in both wrist extensors in all conditions.
- However, a complementary analysis of the electromyographic median frequency of the extensor carpi ulnaris suggested a tendency for pronounced muscle fatigue in the older group when cognitive demands are higher at the lower muscular load level.
- Since the findings may point towards a higher work-related risk of musculoskeletal diseases in older workers associated with cognitive load, further research is needed to verify the relationship between age and cognitive load and determine its significance.

## Supplemental Material

Supplemental Material - Wrist Extensor Muscle Fatigue During a Dual Task With Two Muscular and Cognitive Load Levels in Younger and Older Adults Supplemental Material for Wrist Extensor Muscle Fatigue During a Dual Task With Two Muscular and Cognitive Load Levels in Younger and Older Adults by Florestan Wagenblast, Thomas Läubli, Robert Seibt, Monika A. Rieger and Benjamin Steinhilber in Human Factors
