# Supplementary material for: Wrist Extensor Muscle Fatigue During a Dual Task With Two Muscular and Cognitive Load Levels in Younger and Older Adults
Source: Hum Factors. 2023 Dec 6;66(11):2433–50. doi: 10.1177/00187208231218196 (PMC11453032; doi:10.1177/00187208231218196)
Supplement: Supplemental Material - Wrist Extensor Muscle Fatigue During a Dual Task With Two Muscular and Cognitive Load Levels in Younger and Older Adults [file sj-pdf-1-hfs-10.1177_00187208231218196.pdf]

Supplementary Materials

Supplementary material 1. Mixed analysis of variance applied to perceived muscular and mental effort.

Effect on perceived efforts	Muscular			Mental		
	***df*, *df* residuals**	***F*-values**	***p*-values**	***df*, *df* residuals**	***F*-values**	***p*-values**
Age group	1, 46.0	1.8	0.186	1, 46.0	14.6	< .001[a]
Muscular load	1, 134.8	129.6	< .001[a]	1, 137.7	1.2	0.280
Cognitive load	1, 134.8	3.0	0.085	1, 137.7	330.4	< .001[a]
Age group*muscular load	1, 134.8	45.3	< .001[a]	1, 137.7	0.1	0.804
Age group*cognitive load	1, 134.8	0.3	0.609	1, 137.7	19.8	< .001[a]
Muscular load*cognitive load	1, 134.8	1.1	0.290	1, 137.7	< .1	0.848
Age group*muscular load*cognitive load	1, 134.8	1.4	0.249	1, 137.7	3.5	0.064

Results of the 3-factor mixed analyses of variance applied to muscular and mental effort ratings after each dual-task condition considering the factors age group (older, younger), muscular load (5%MVC, 10%MVC), and cognitive load (0-back, 2-back task).

[a] Statistically significant effect ($\alpha = 0.05$).

Supplementary material 2. Perceived muscular and mental effort.

Perceived effort	Age group	5%MVC		10%MVC	
		**0-back task**	**2-back task**	**0-back task**	**2-back task**
Muscular	Older	5.21 (2.30)	5.58 (2.36)	5.96 (2.37)	6.33 (2.20)
	Younger	3.33 (1.24)	3.92 (1.86)	6.67 (1.63)	6.45 (1.74)
Mental	Older	5.46 (2.21)	7.41 (1.91)	5.25 (2.45)	7.88 (1.75)
	Younger	2.58 (1.47)	6.63 (1.81)	3.08 (1.50)	6.58 (1.82)

Means and standard deviations in brackets for muscular and mental effort assessment after each dual-task condition depending on muscular (5%MVC, 10%MVC) and cognitive load levels (0-back, 2-back task) in the older and younger age group.

Supplementary material 3. Force steadiness of the tracking task.

Age group	Time course	5%MVC		10%MVC	
		**0-back task [N]**	**2-back task [N]**	**0-back task [N]**	**2-back task [N]**
Older	block1	0.300 (0.135)	0.348 (0.113)	0.430 (0.306)	0.442 (0.195)
	block2	0.340 (0.199)	0.338 (0.127)	0.416 (0.265)	0.426 (0.201)
	block3	0.315 (0.123)	0.342 (0.137)	0.492 (0.471)	0.477 (0.204)
	block5	0.359 (0.152)	0.416 (0.183)	0.486 (0.289)	0.491 (0.225)
	block6	0.349 (0.189)	0.356 (0.115)	0.496 (0.326)	0.527 (0.288)
Younger	block1	0.258 (0.062)	0.281 (0.105)	0.350 (0.119)	0.366 (0.162)
	block2	0.260 (0.073)	0.287 (0.084)	0.379 (0.132)	0.411 (0.213)
	block3	0.278 (0.075)	0.297 (0.106)	0.387 (0.127)	0.432 (0.203)
	block5	0.311 (0.117)	0.326 (0.150)	0.423(0.153)	0.470 (0.251)
	block6	0.291 (0.085)	0.322 (0.095)	0.461 (0.176)	0.475 (0.208)

Means and standard deviations in brackets for time course (block1 to block6) of force steadiness of the tracking task during each dual task condition depending on muscular (5%MVC, 10%MVC) and cognitive load levels (0-back, 2-back task) in the older and younger age group. Block4 is excluded due to potential bias from MVC measurements after block3.

Supplementary material 4. Mixed analyses of variance applied to force steadiness.

**Effect on force steadiness**	**Force steadiness**		
	***df*, *df* residuals**	***F*-values**	***p*-values**
Age group	1, 46.0	2.0	0.169
Muscular load	1, 319.1	84.5	< .001[a]
Cognitive load	1, 319.1	4.5	0.034[a]
Time course	1, 275.0	0.6	0.440
Age group*muscular load	1, 319.1	13.1	< .001[a]
Age group*cognitive load	1, 319.1	0.1	0.702
Age group*time course	1, 275.0	0.7	0.399
Muscular load*cognitive load	1, 319.1	0.1	0.794
Muscular load*time course	1, 275.0	5.8	0.017[a]
Cognitive load*time course	1, 275.0	< .1	0.879
Age group*muscular load*cognitive load	1, 319.1	< .1	0.923
Age group*muscular load*time course	1, 275.0	0.3	0.617
Age group*cognitive load*time course	1, 275.0	0.1	0.787
Muscular load*cognitive load*time course	1, 275.0	0.3	0.603
Age group*muscular load*cognitive load*time course	1, 275.0	0.5	0.483

Results of the 4-factor mixed analyses of variance applied to force steadiness (SD) considering the factors age group (older, younger), muscular load (5%MVC, 10%MVC), cognitive load (0-back, 2-back task), and time course (block1, block6)

[a] Statistically significant effect ($\alpha = 0.05$).

Supplementary material 5. Maximum voluntary contraction force.

Age group	Time course	5%MVC		10%MVC	
		**0-back task [N]**	**2-back task [N]**	**0-back task [N]**	**2-back task [N]**
Older	start	127.9 (43.8)	134.8 (56.9)	129.2 (53.7)	133.5 (49.4)
	midpoint	133.1 (47.2)	132.4 (48.4)	124.3 (43.5)	126.4 (47.1)
	end	126.8 (48.0)	127.6 (54.7)	113.3 (45.3)	121.4 (48.0)
Younger	start	143.5 (48.2)	140.4 (47.7)	146.7 (52.1)	142.2 (42.2)
	midpoint	145.1 (47.4)	141.5 (47.6)	138.9 (50.3)	133.6 (40.7)
	end	143.0 (45.1)	138.0 (48.2)	133.3 (48.7)	123.9 (39.4)

Means and standard deviations in brackets for time course (start, midpoint, end) of the maximum voluntary contraction force during each dual-task condition depending on muscular (5%MVC, 10%MVC) and cognitive load levels (0-back, 2-back task) in the older and younger age group.

Supplementary material 6. Median frequency of the extensor carpi ulnaris.

**Age group**	**Time course**	**5%MVC**		**10%MVC**	
		**0-back task [Hz]**	**2-back task [Hz]**	**0-back task [Hz]**	**2-back task [Hz]**
Older	block1	104.4 (11.9)	107.6 (14.9)	106.2 (10.6)	105.0 (12.5)
	block2	103.0 (11.5)	100.5 (10.4)	103.4 (10.2)	101.1 (13.8)
	block3	102.2 (12.4)	100.9 (9.3)	103.7 (12.0)	102.4 (11.4)
	block5	104.6 (13.2)	99.4 (12.3)	104.9 (11.4)	101.2 (12.0)
	block6	102.1 (12.0)	99.3 (12.1)	104.3 (12.3)	102.3 (12.5)
Younger	block1	107.4 (23.1)	108.1 (19.1)	110.5 (20.8)	108.0 (21.2)
	block2	105.7 (22.6)	107.3 (20.3)	109.4 (21.8)	107.5 (21.1)
	block3	105.1 (21.9)	107.3 (18.5)	109.0 (21.3)	107.9 (21.9)
	block5	105.0 (23.6)	105.6 (18.2)	108.0 (19.7)	107.9 (20.7)
	block6	104.8 (22.4)	105.9 (19.5)	108.1 (18.9)	107.7 (20.0)

Means and standard deviations in brackets for time course (block1 to block6) of the median frequency of the electromyographical muscle activity of the extensor carpi ulnaris during each dual-task condition depending on muscular (5%MVC, 10%MVC) and cognitive load levels (0-back, 2-back task) in the older and younger age group. Block4 is excluded due to potential bias from MVC measurements after block3.

Supplementary material 7. Development of median frequency of the extensor carpi ulnaris.

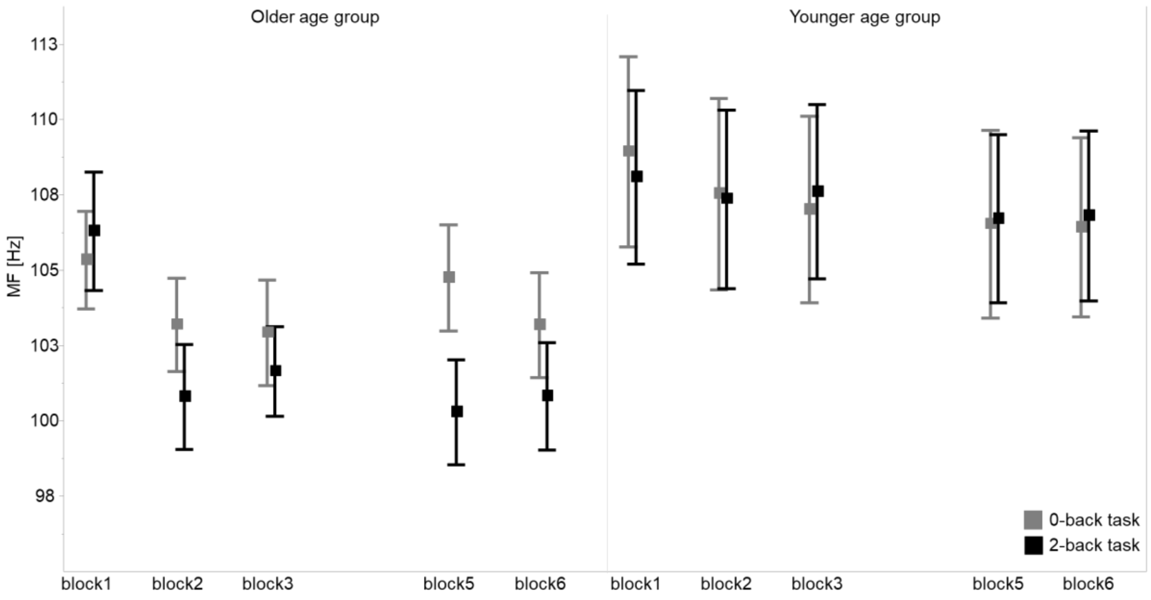

Means and standard errors of the median frequency (MF) of the extensor carpi ulnaris measured by surface electromyography during the tracking task from block1 to block6 comparing cognitive load conditions (0-back task grey, 2-back task black) with respect to age groups (older left, younger right). Block4 is excluded due to potential bias from MVC measurements after block3.

Supplementary material 8. Root mean square of the extensor carpi ulnaris.

Age group	Time course	5%MVC		10%MVC	
		**0-back task [% MVE]**	**2-back task [% MVE]**	**0-back task [% MVE]**	**2-back task [% MVE]**
Older	block1	13.3 (4.2)	13.3 (5.6)	18.5 (7.0)	18.7 (8.1)
	block2	13.9 (5.1)	13.0 (7.0)	18.7 (8.5)	19.7 (9.5)
	block3	14.0 (5.7)	13.1 (6.3)	19.4 (9.6)	19.9 (9.7)
	block5	16.0 (6.3)	14.6 (9.0)	20.7 (9.1)	19.8 (9.2)
	block6	15.2 (6.8)	14.4 (8.7)	21.7 (9.2)	20.6 (9.9)
Younger	block1	12.1 (6.3)	12.7 (6.3)	17.2 (8.5)	17.5 (10.1)
	block2	11.6 (6.4)	13.4 (6.9)	16.5 (7.7)	17.6 (10.1)
	block3	12.1 (6.6)	14.0 (6.9)	16.9 (8.0)	17.8 (10.6)
	block5	12.1 (5.9)	14.5 (6.4)	17.3 (8.1)	18.4 (9.1)
	block6	12.6 (6.2)	14.3 (6.7)	17.4 (8.2)	18.7 (9.1)

Means and standard deviations in brackets for time course (block1 to block6) of the normalized median root mean square of the electromyographical muscle activity of the extensor carpi ulnaris during each dual-task condition depending on muscular (5%MVC, 10%MVC) and cognitive load levels (0-back, 2-back task) in the older and younger age group. Block4 is excluded due to potential bias from MVC measurements after block3.

Supplementary material 9. Development of root mean square of the extensor carpi ulnaris.

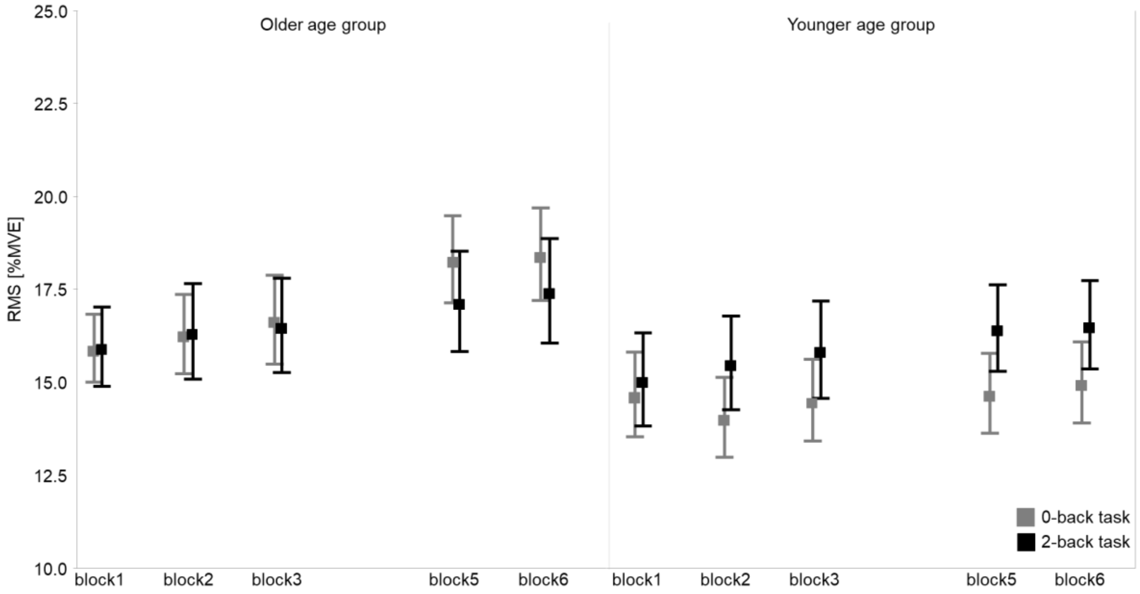

Means and standard errors of the normalized root mean square (RMS) of the extensor carpi ulnaris measured by surface electromyography during the tracking task from block1 to block6 comparing cognitive load conditions (0-back task grey, 2-back task black) with respect to age groups (older left, younger right). Block4 is excluded due to potential bias from MVC measurements after block3.

Supplementary material 10. Four-factor mixed analyses of variance applied to median frequency and root mean square of the extensor digitorum.

**Effect on muscle activity of the extensor digitorum**	**MF**			**RMS**		
	***df, df* residuals**	***F*-values**	***p*-values**	***df, df* residuals**	***F*-values**	***p*-values**
Age group	1, 46.0	5.3	0.027[a]	1, 46.0	0.6	0.425
Muscular load	1, 315.3	0.3	0.591	1, 321.1	100.2	< .001[a]
Cognitive load	1, 315.3	0.2	0.639	1, 321.1	1.1	0.302
Time course	1, 274.1	15.1	< .001[a]	1, 274.5	26.8	< .001[a]
Age group*muscular load	1, 315.3	< .1	0.764	1, 321.1	0.2	0.618
Age group*cognitive load	1, 315.3	0.1	0.711	1, 321.1	< .1	0.911
Age group*time course	1, 274.1	2.4	0.123	1, 274.5	1.5	0.228
Muscular load*cognitive load	1, 315.3	0.2	0.631	1, 321.1	1.7	0.193
Muscular load*time course	1, 274.1	0.1	0.721	1, 274.5	0.2	0.665
Cognitive load*time course	1, 274.1	1.8	0.184	1, 274.5	0.3	0.605
Age group*muscular load*cognitive load	1, 315.3	0.3	0.592	1, 321.1	< .1	0.815
Age group*muscular load*time course	1, 274.1	< .1	0.893	1, 274.5	0.5	0.480
Age group*cognitive load*time course	1, 274.1	< .1	0.857	1, 274.5	0.8	0.366
Muscular load*cognitive load*time course	1, 274.1	< .1	0.949	1, 274.5	< .1	0.872
Age group*muscular load*cognitive load*time course	1, 274.1	0.2	0.662	1, 274.5	< .1	0.993

Results of the of the electromyographical muscle activity of the extensor digitorum parameterized as the median frequency (MF) and normalized root mean square (RMS) considering the factors age group (older, younger), muscular load (5%MVC, 10%MVC), cognitive load (0-back, 2-back task), and time course (block1, block6).

[a] Statistically significant effect ($\alpha = 0.05$).

Supplementary material 11. Median frequency of the extensor digitorum.

Age group	Time course	5%MVC		10%MVC	
		**0-back task [Hz]**	**2-back task [Hz]**	**0-back task [Hz]**	**2-back task [Hz]**
Older	block1	88.6 (14.2)	90.9 (17.4)	90.2 (13.7)	88.5 (14.6)
	block2	87.1 (12.6)	87.7 (15.2)	90.8 (16.4)	86.6 (14.0)
	block3	87.4 (14.2)	87.4 (15.4)	89.4 (14.2)	86.9 (14.6)
	block5	86.9 (13.9)	88.5 (18.4)	89.5 (14.5)	85.8 (13.4)
	block6	86.2 (12.8)	86.7 (14.0)	87.8 (12.8)	85.4 (13.4)
Younger	block1	81.2 (10.2)	82.3 (9.3)	79.9 (9.4)	81.6 (14.2)
	block2	81.0 (10.8)	81.7 (9.8)	80.4 (9.9)	81.0 (14.9)
	block3	79.1 (9.5)	81.6 (9.7)	81.0 (10.9)	80.1 (13.9)
	block5	80.3 (9.8)	81.7 (11.6)	80.1 (10.7)	79.7 (14.6)
	block6	80.5 (10.5)	80.2 (11.2)	79.8 (10.7)	79.3 (14.9)

Means and standard deviations in brackets for time course (block1 to block6) of the median frequency of the electromyographical muscle activity of the extensor digitorum during each dual-task condition depending on muscular (5%MVC, 10%MVC) and cognitive load levels (0-back, 2-back task) in the older and younger age. Block4 is excluded due to potential bias from MVC measurements after block3.

Supplementary material 12. Root mean square of the extensor digitorum.

Age group	Time course	5%MVC		10%MVC	
		0-back task [% MVE]	2-back task [% MVE]	0-back task [% MVE]	2-back task [% MVE]
Older	block1	10.6 (5.6)	10.6 (6.3)	14.4 (7.5)	15.2 (7.5)
	block2	11.0 (6.0)	11.1 (6.4)	14.5 (8.2)	15.8 (7.8)
	block3	11.0 (6.6)	11.5 (6.9)	14.4 (8.0)	16.3 (7.9)
	block5	12.5 (6.4)	11.4 (6.9)	16.3 (7.6)	17.5 (7.8)
	block6	12.5 (6.9)	12.3 (7.4)	17.3 (7.7)	17.7 (8.2)
Younger	block1	10.0 (4.9)	9.4 (4.6)	13.9 (5.0)	14.4 (7.1)
	block2	9.9 (4.9)	10.3 (4.8)	13.7 (4.6)	14.8 (7.1)
	block3	9.7 (4.4)	10.6 (4.7)	14.3 (4.8)	14.9 (7.2)
	block5	10.6 (4.5)	11.0 (4.5)	15.1 (6.5)	15.9 (7.5)
	block6	10.9 (4.8)	11.5 (5.0)	14.7 (5.3)	16.1 (7.4)

Means and standard deviations in brackets for time course (block1 to block6) of the normalized median root mean square of the electromyographical muscle activity of the extensor digitorum during each dual-task condition depending on muscular (5%MVC, 10%MVC) and cognitive load levels (0-back task, 2-back task) in the older and younger age group. Block4 is excluded due to potential bias from MVC measurements after block3.
